# Identification and Validation of Tryptophan Metabolism–Related Genes in Diabetic Kidney Disease and Construction of a Clinical Prediction Model

**DOI:** 10.1155/jdr/2736801

**Published:** 2025-05-08

**Authors:** Shaojie Liu, Qingqing Jiang, Wenli Li, Jinbao Shi, Binxuan Wu, Man Xiong, Liuying Huang

**Affiliations:** Department of Nephrology, Blood Purification Research Centre, Ningde Hospital of Traditional Chinese Medicine, Fujian University of Traditional Chinese Medicine, Ningde, China

**Keywords:** biomarkers, diabetic kidney disease, tryptophan metabolism

## Abstract

**Background:** Diabetic kidney disease (DKD) is a common microvascular complication of diabetes mellitus (DM). Amino acid (AA) homeostasis has an important impact on renal hemodynamics and glomerular hyperfiltration in patients with DKD, and the metabolite level of tryptophan (TRP), an AA, has been associated with various diseases.

**Methods:** In this study, DKD tubule- and glomerulus-related microarray datasets were collected from the GEO database, and DKD-related modular genes were identified by weighted gene coexpression network analysis (WGCNA). TRP metabolism–related genes (TRGs) were downloaded from the MSigDB database, and the key genes were obtained by taking the intersection of DKD differentially expressed genes, TRGs, and modular genes. Validated with the Nephrseq v5 database and performed clinical prediction model construction. The association of pivotal genes with immune cell infiltration was verified using CIBERSORTx software. The protein expression of the key genes was verified by qPCR, Western blot, immunohistochemistry, and immunofluorescence.

**Results:** Four hundred and seventy seven DEGs were identified in the GSE30529 dataset, 392 DEGs were identified in the GSE30528 dataset, and the intersection of the DEGs in the two datasets, the module with the most significant correlation with DKD obtained by WGCNA, and the TRGs were taken, respectively. Five key genes were finally obtained (AOC1, HAAO, STAT1, OGDHL, and TDO2). Compared with control-group mice, the expression of AOC1, HAAO, and OGDHL was significantly downregulated, and the expression of STAT1 and TDO2 was significantly elevated in DKD mice. The diagnostic model was constructed using the key genes AUC = 0.996.

**Conclusion:** Our study suggests that the AOC1, HAAO, and STAT1 genes may be potential diagnostic biomarkers of tubular injury in DKD. OGDHL and TDO2 may be potential diagnostic biomarkers of glomerular injury in DKD. The model constructed using AOC1, HAAO, STAT1, OGDHL, and TDO2 had good disease differentiation.

## 1. Introduction

Diabetes mellitus (DM) affects about 10% of the population worldwide [1]. Diabetic kidney disease (DKD), the most common microvascular complication of DM, has a grim picture, with approximately 40% of diabetic patients ultimately developing DKD [2]. The pathomechanisms of DKD are complex and varied, mainly involving activation of the renin-angiotensin-aldosterone system (RAAS), accumulation of end products of advanced glycosylation, epithelial–mesenchymal transition, inflammatory response, cellular stress, apoptosis, focal death, and autophagy, among others [3]. In recent years, studies have shown [2] that amino acid (AA) homeostasis is important for renal haemodynamic responses and glomerular hyperfiltration in DM patients and that disturbances in AA metabolic homeostasis can lead to abnormal accumulation of deleterious metabolites or activation of metabolic enzymes, which may trigger cellular signaling during the progression of DKD, such as oxidative stress, inflammation, fibrosis, and apoptosis. Therefore, AA is a biomarker of DKD progression and an important pathogenic factor.

Tryptophan (TRP), an essential AA for protein synthesis, is found in low levels in cells and proteins, can only be obtained through the dietary route, and is a substrate for various bioactive compounds with important physiological roles [4]. A correlation between plasma TRP and the rapid progression of DKD has been reported [5]. TRP metabolism involves several pathways, the main ones of which include the kynurenine (Kyn) pathway, the serotonin (5-HT) pathway, and the indole pathway. These pathways are responsible for the catabolism and transformation of TRP, allowing it to generate various metabolites that contribute to TRP's overall metabolism [6]. Zhang et al.'s [7] study demonstrated the association between TRP metabolic pathways and the progression of DKD and that the metabolite of TRP, indole acetic acid (IAA), was positively correlated with the deterioration of renal function. The Kyn pathway, as an important pathway of TRP metabolism, is a renal disease prevention and treatment. A promising target, the disordered catabolism of the TRP Kyn pathway and the indole pathway, has been demonstrated to be closely associated with cerebrovascular disease (CVD) in chronic kidney disease (CKD) patients [8].

DKD leads to reduced renal function, which causes the accumulation of TRP metabolites and accelerates the progression of acute kidney injury (AKI) or CKD [9]. Circulating levels of various metabolites are altered at the onset of kidney injury, with metabolites associated with the TRP metabolic pathway significantly altered, in addition to the common uremic toxins, acylcarnitines, etc. [10]. Various studies have assessed the impact of the toxic properties of metabolites accumulated by the Kyn pathway and found that these derivatives bind to insulin to form excitotoxic complexes that may lead to systemic disorders, including insulin resistance, altered blood pressure, and renal injury [11]. High serum expression of 5-HT and its end-product metabolite (5-hydroxyindoleacetic acid) is closely related to the pathogenesis of DKD [12]. Therefore, it can be hypothesised that TRP metabolism metabolites are involved in the progression of DKD. However, there is currently a lack of clarity regarding the mechanisms involved in TRP metabolism in the progression of DKD. The present study is planned to investigate the role of TRGs in the pathogenesis of DKD using bioinformatics analysis, aiming to provide new targets and ideas for preventing the progression of DKD, thus supporting more precise and individualised treatment.

## 2. Materials and Methods

### 2.1. Microarray Data Processing

The DKD renal tubular microarray dataset GSE30529 (GPL571) was collected from the GEO database, including 10 DKD patients and 12 control samples, and the DKD glomerular microarray dataset GSE30528 (GPL571) was collected, including 9 DKD patients and 13 control samples. The above two microarray datasets were used as the training set for this experiment. The inclusion criteria for patients were a clinical diagnosis of DM, a positive urine test paper, or an elevated urine protein/creatinine ratio, and renal biopsy histology consistent with the characteristics of DKD. In contrast, patients with comorbidities of other renal diseases, systemic immune disorders, and biopsy findings of other pathological changes were excluded. The training dataset was standardised using the “limma package” before analysis (Table 1).

In addition, the DKD tabular dataset GSE104954 (GPL22945) and the DKD whole kidney biopsy specimen dataset GSE142025 (GPL20301) were collected from the GEO database, including 34 DKD samples and 27 control samples as the validation set, which was standardised with the “sva” package to remove batch effects and normalised with the “limma” package before analysing the data (Table 1).

In this study, the TRGs were obtained from the MSigDB database [13], including KEGG_TRYPTOPHAN_METABOLISM, REACTOMETRYPTOPHANCATABOLI, and WPTRY PTOPHAN_METABOLISM. After removing duplicated genes, 51 TRGs were included in this study (Table S1).

### 2.2. Weighted Gene Coexpression Network Analysis (WGCNA)

Gene coexpression networks were constructed using the R package called “WGCNA.” The adjacency matrix consists of weighted correlation coefficients. Subsequently, the adjacency matrix was converted to a topological overlap matrix (TOM). A soft threshold power of 10 and a minimum module size 300 were set to filter the core modules. The modules were then tested using the Pearson correlation test with a significance threshold of *p* < 0.05.

### 2.3. Identification of Differentially Expressed Genes

To find the DEGs between DKD and healthy samples, “limma” R package was used. The cut-off criteria were adjusted for *p* < 0.05 and log|FC| > 1. Using the “ggplots” package, the distribution of the differential genes was presented in volcano plots.

### 2.4. DKD-Associated Glomerular and Tubular Genes Take Intersection With TRG-Associated Genes

We took intersections of TRGs with DKD-related module genes and DKD differentially expressed genes from WGCNA, respectively. Venn plots were used to describe the details of the overlapping genes.

### 2.5. Construction and Validation of Clinical Prediction Models

Separately, the five key genes associated with TRGs were used to calculate column line plot scores using the R (4.3.2) “nomogramFormula” package, and the column line plots were used to predict the occurrence of DKD. We performed a receiver operating characteristic (ROC) curve analysis to assess model performance using the “pROC” package for R [14].

### 2.6. Immune Cell Infiltration Analysis

Immune cell infiltration levels were estimated using the CIBERSORT [15] algorithm and the LM22 feature matrix, combined with the human feature gene matrix to exclude data with immune cell enrichment scores > 0. The correlation between immune cells and TRGs was determined by combining the gene expression matrix of the DKD dataset, and the correlation heatmap was plotted using the R package “pheatmap.” The Wilcoxon rank sum test assessed differences in immune cell infiltration between the DKD and control groups.

### 2.7. Clinical Data Validation and Diagnostic Efficacy of Hub Genes in DKD Assessment

Information of hub genes associated with TRGs was downloaded from the Nephrseq online open-access platform (http://v5.nephroseq.org) to compare the mRNA expression levels of hub genes in DKD samples and controls.

### 2.8. Experimental Animals

Six 8-week-old BKS-DB(ko/ko) mice were selected as the experimental group, and six 8-week-old BKS/m mice were selected as the control group. All mice were purchased from Guangdong Jicui Pharmachem Biotechnology Co., Ltd. and kept in the pathogen-free barrier environment animal house of Fujian University of Traditional Chinese Medicine. All animal care and experimental procedures were approved by the Animal Ethics Committee of Fujian University of Traditional Chinese Medicine (Ethical Review Approval Number: FJTCM IACUC 3 W2024041). After 2 weeks of adaptive rearing, fasting blood glucose ≥ 16.7 mmol/L in tail vein blood was measured for three consecutive days, suggesting successful model preparation. After continued rearing for 14 weeks, the mice were killed by neck-breaking, and bilateral kidney tissues were taken for subsequent experiments.

### 2.9. qPCR

Total RNA was extracted from the samples with the Vazyme Total RNS Extraction Kit, RNA reverse transcription was performed with the NovoScriptPlus All-in-one 1st Strand cDNA Synthesis SuperMix Kit, and qPCR amplification was performed with the NovoStart SYBR qPCR SuperMix Plus kit for qPCR amplification. All primers were designed and synthesised by Aashan Bio, and the primer sequences are shown in Table S2. mRNA levels of the target genes were calculated using the 2^−ΔΔCt^ method. The mRNA levels of the target genes were calculated using the 2^−ΔΔCt^ method. mRNA levels of the target genes were normalised to *β*-actin.

### 2.10. Immunohistochemistry

Paraffin sections of mouse kidney tissue were deparaffinised, hydrated with gradient ethanol, washed with double steam water, and repaired with microwave antigen. Next, endogenous peroxidase was sequestered with 3% H_2_O_2_, followed by 3% bovine serum albumin. AOC1 (amine oxidase copper content 1) (1:200; Cloud-Clone Corp, PAA656Mu02), HAAO (1:200; immunoway, YN0362), STAT1 (1:200; immunoway, YT4439), OGDHL (1:200; immunoway, YT8128), and TDO2 (1:200; immunoway, YN5825) were used for the antigen repair. The samples were incubated in a refrigerator at 4°C overnight. Then, the secondary antibody was added, and another incubation period of 50 min was performed. Diaminobenzidine was added for colour development, and hematoxylin was added for nuclei staining. The samples were observed under a light microscope and photographed for storage.

### 2.11. Western Blot

Tissues and cells were lysed with RIPA lysis buffer (Solarbio, Beijing, China). These lysates were then collected by quantifying the protein concentration with the Instant BCA Protein Assay Kit (Beyotime, Shanghai, China). Primary antibody was used the same way as the IHC section. Signal was measured by measuring the signal with the Enhanced Chemiluminescence Substrate (Thermo Fisher Scientific, Waltham, Massachusetts, United States).

### 2.12. Immunofluorescence

Mouse kidney tissue paraffin sections were deparaffinised, microwaved for antigen repair, treated with 0.3% Triton X-100 for membrane breaking for 10 min, subjected to dropwise addition of sample containment solution, and incubated in a wet box at room temperature for 2 h, and the containment solution was removed. Add primary antibody, nephrin (1:100; PROGEN, GP-N2), the rest of the primary antibody was used the same way as the IHC part, and the wet box was incubated at 4°C overnight. After removing the wet box and rewarming for 30 min, the working solution of the secondary antibody was prepared and added dropwise at the ratio of 1:200 (taking care to avoid light), incubated at 37°C for 2 h, sealed with antifluorescence burst sealer containing DAPI, observed under a confocal microscope, and photographed.

## 3. Results

### 3.1. Data Preprocessing

**Figure 1:**
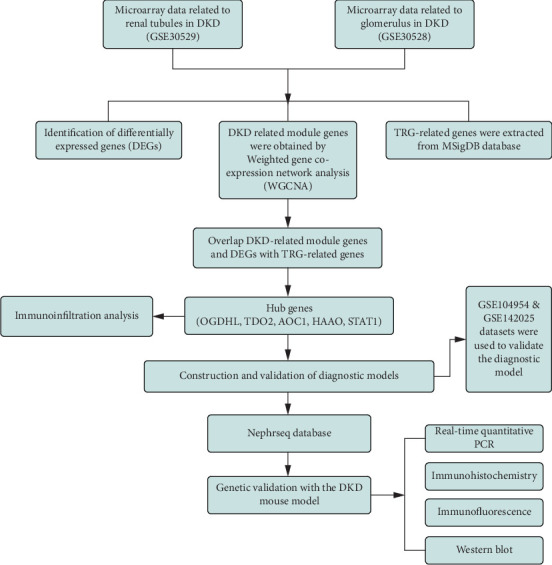
Flowchart of the overall analysis in this study.

The overall flowchart of the analysis is shown in Figure 1. In this study, DKD renal tubular microarrays were collected from the GSE30529 dataset, and DKD glomerular microarrays were collected from the GSE30528 dataset; the above two datasets were used as the training set for this experiment, and the data were standardised using the “limma” package before data analysis. In addition, the DKD tubule-related dataset GSE104954 (GPL22945) and the DKD whole kidney biopsy specimen dataset GSE142025 (GPL20301) were collected from the GEO database, including 34 DKD samples and 27 control samples in total, which served as the validation set. Before data analysis, we applied the “sva” package to remove batch effects and the “limma” package to standardise the data.

### 3.2. Analysis of Weighted Coexpression Networks and Identification of Core Modules

**Figure 2:**
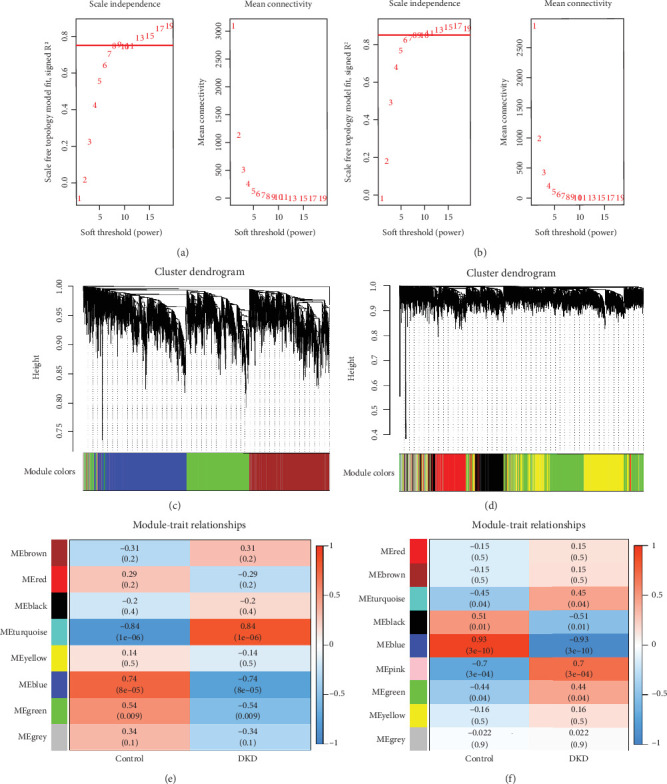
Network construction and module detection in GSE30528 and GSE30529 using WGCNA. (a) Select soft threshold power, and perform network connectivity analysis with WGCNA in the GSE30529 dataset. (b) Select soft threshold power, and perform network connectivity analysis with WGCNA in the GSE30528 dataset. (c) Cluster dendrogram in the GSE30529 dataset. (d) Cluster dendrogram graph in the GSE30528 dataset. (e) Heatmap of module–trait relationships between db/db groups and db/m groups in the GSE30529 dataset. (f) Heatmap of module–trait relationships for group db/db versus group db/m in the GSE30528 dataset.

Scale-free networks were constructed with the soft threshold set to 10 (Figure 2a,b). Then, the adjacency matrix and TOM were built. We then calculated the module eigengenes representing each module's overall gene expression level; these were clustered according to their correlation (Figure 2c,d). We analysed the correlation of each eigengene with the phenotype (DKD or control samples). We selected the two modules with the most significant correlation with DKD in the tubule- and glomerulus-related datasets, respectively (Figure 2e,f).

In the tubule-related dataset, we picked the turqupise module (cor = 0.84, *p* = 1e − 06) and the blue module (cor = −0.74, *p* = 8e − 05). For the glomerulus-related dataset, we picked the blue module (cor = −0.93, *p* = 3e − 10) and the pink module (cor = 0.7, *p* = 3e − 04).

### 3.3. Identification of Differentially Expressed Genes in the Control Group and the DKD Group

**Figure 3:**
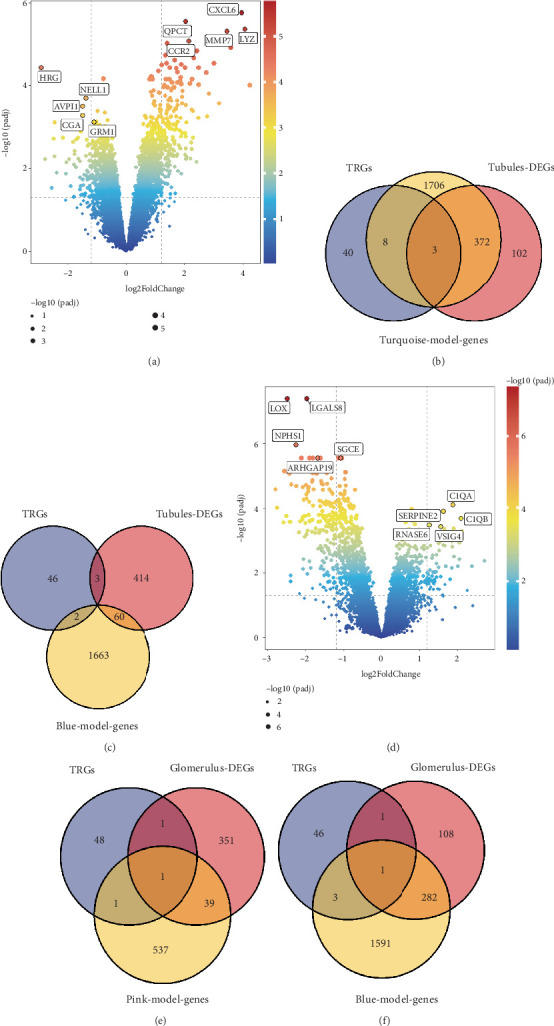
Differentially expressed gene analysis of the DKD and TRG datasets. (a) Volcano plot of DEGs in the GSE30529 dataset. (b, c) Venn diagram of DEGs in the GSE30529 dataset. (d) Volcano plots of DEGs in the GSE30528 dataset. (e, f) Venn diagrams of DEGs in the GSE305298 dataset. Blue represents the TRG group, pink represents the diabetes group, and yellow represents the modular genes. Co-DEGs are common differentially expressed genes.

Four hundred and seventy-seven DEGs were identified in the GSE30529 dataset, with 324 upregulated and 153 downregulated genes. Three hundred and ninety-two DEGs were identified in the GSE30528 dataset, of which 112 were upregulated genes and 280 downregulated genes. The top five upregulated DEGs and the top five downregulated DEGs in both datasets were found by selecting the columns of log–fold changes in ascending order from the results of the variance analysis and are shown in the volcano plots (Figure 3a,d).

### 3.4. DKD-Related Modular Genes, DKD Differentially Expressed Genes, and TRG-Related Gene Overlap

TRGs were downloaded from the MSigDB database, and we overlapped these genes with DKD-related module genes and DKD differentially expressed genes from WGCNA, respectively. Venn plots were used to describe the details of the overlapping genes. Five key genes related to TRP metabolism, that is, AOC1, HAAO, STAT1, OGDHL, and TDO2, were finally obtained.

### 3.5. Construction and Validation of Diagnostic Models

**Figure 4:**
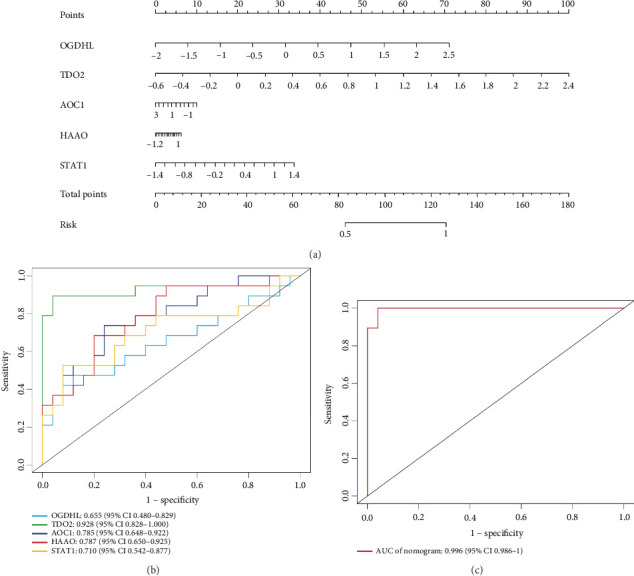
Clinical predictive models constructed from training dataset. (a) Diagnostic curve analysis of pivotal genes. (b) Working characteristic curve analysis of subjects for the pivotal genes. (c) Working characteristic curve analysis of subjects with five pivotal genes in common.

Clinical prediction models were constructed using the key genes described above and presented in a column–line graph (Figure 4a). OGDHL and TDO2 had longer straight lines in the column–line plot, which we hypothesised might be valuable predictors for diagnosing DKD progression. The combined model AUC = 0.996 (95% CI: 0.986, 1) > 0.85, which we considered to be well discriminated and superior to the single-part predictive model described above (Figure 4b,c).

**Figure 5:**
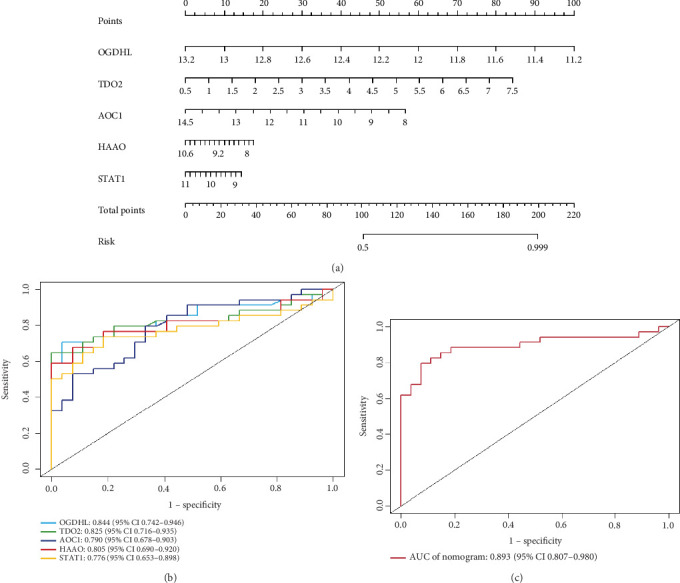
Clinical predictive models constructed from validation dataset. (a) Diagnostic curve analysis of pivotal genes. (b) Working characteristic curve analysis of subjects for the pivotal genes. (c) Working characteristic curve analysis of subjects with five pivotal genes in common.

The diagnostic model was validated in the GSE104954 and GSE142025 validation datasets, which predicted a model with AUC = 0.893 > 0.85. Thus, we still considered the model well discriminated (Figure 5).

### 3.6. Immune Infiltration Analysis of DKD Datasets

**Figure 6:**
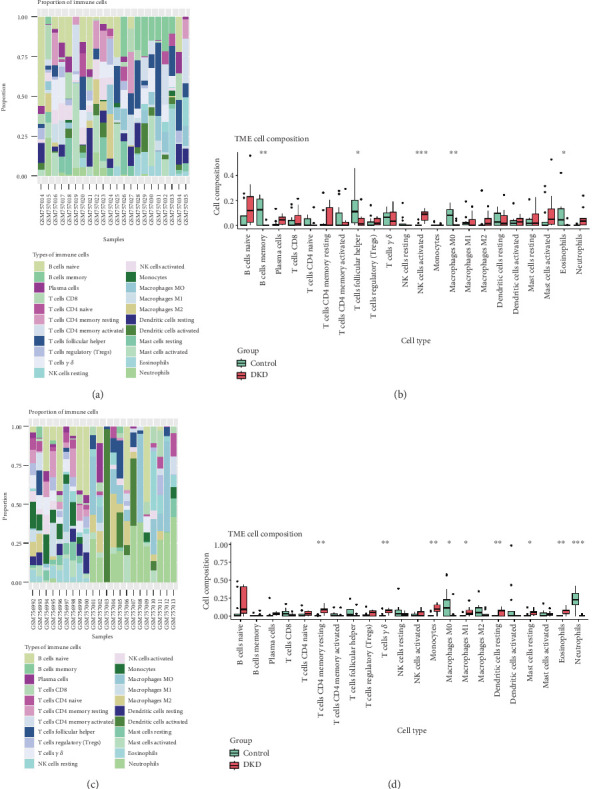
Immune infiltration analysis of immune cell types in the GSE30529 and GSE305298 dataset samples. (a) Correlation of immune cells with expression profiling data in the healthy and DKD groups in the GSE30529 dataset. (b) Differences in the distribution of memory B cells, helper regulatory T cells, activated NK cells, M0 macrophages, and eosinophils between the healthy and DKD groups in the GSE30529 dataset. (c) Correlation of immune cells with expression profiling data in the healthy and DKD groups in the GSE305298 dataset. (d) Differences in the distribution of resting CD4+ T cells, *γ δ* T cells, monocytes, M0 macrophages, M1 macrophages, resting dendritic cells, resting mast cells, eosinophils, and neutrophils between the healthy and DKD groups in the GSE305298 dataset. ⁣^∗^*p* < 0.05, ⁣^∗∗^*p* < 0.01, and ⁣^∗∗∗^*p* < 0.001.

The correlation between immune cells and expression profiling data was calculated for the healthy and DKD groups in both datasets, and the infiltration of 22 immune cell types in each sample was plotted as a bar graph in the GSE30529 (Figure 6a) and GSE30528 (Figure 6c) datasets. In the tubule-associated DKD dataset, there were differences in the distribution of memory B cells, helper regulatory T cells, activated NK cells, M0 macrophages, and eosinophils between the healthy and DKD groups (Figure 6b). In the glomerulus-associated DKD dataset, there were differences in the distribution of resting CD4+ T cells, *γδ* T cells, monocytes, M0 macrophages, M1 macrophages, resting dendritic cells, resting mast cells, eosinophils, and neutrophils between the healthy and DKD groups (Figure 6d).

**Figure 7:**
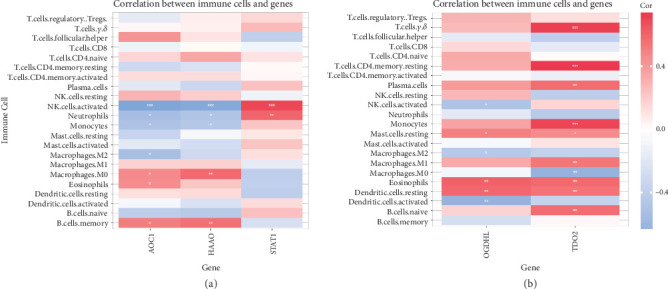
Heatmap of correlation of pivotal genes in the GSE30529 and GSE305298 datasets. (a) Association of three key genes (AOC1, HAAO, STAT1) with the number of immune cells in the GSE30529 dataset. (b) Association of two key genes (OGDHL, TDO2) with the number of immune cells in the GSE305298 dataset.

Correlation heatmap demonstrating the association of three key genes (AOC1, HAAO, and STAT1) in the GSE30529 (Figure 7a) dataset and two key genes (OGDHL and TDO2) in the GSE30528 (Figure 7b) dataset with the number of immune cells. AOC1, HAAO, and STAT1 significantly correlated with activated NK cell infiltration (*p* < 0.001). TDO2 was significantly associated with *γδ* T cells, resting CD4+ T cells, and monocyte infiltration (*p* < 0.001).

### 3.7. Construction of DKD Animal Model

**Figure 8:**
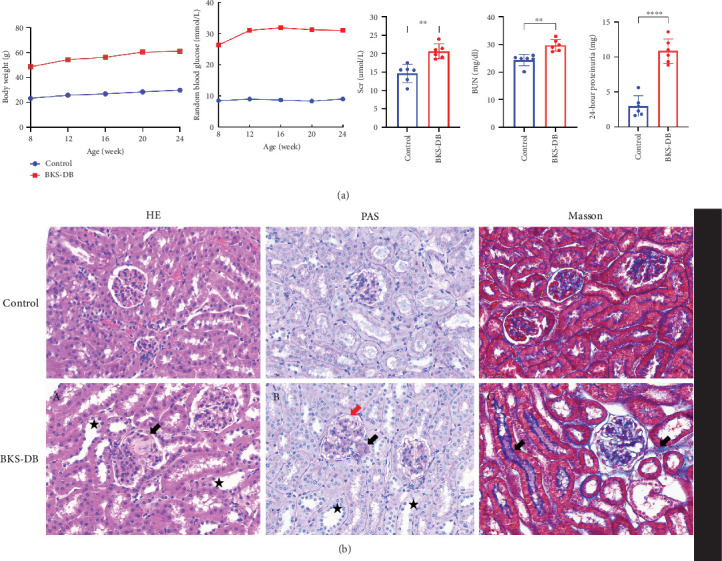
DKD mouse model construction. (a) Changes in blood glucose and body weight and expression levels of creatinine, urea, and 24-h urinary protein in control and BKS-DB mice. (b) HE, PAS, and Masson staining of kidneys of control and BKS-DB mice. Bar: 30 *μ*m. (A) arrow symbol: glomerulosclerosis. ★: Brush border detachment of renal tubular epithelial cells. (B) black arrow symbol: dilatation of capillaries. Red arrow symbol: plasma exudative fibrous cap. ★: brush border detachment of renal tubular epithelial cells. (C) Significant increase in renal tubular and interstitial collagen levels.

Blood glucose and body weight were monitored at 4-week intervals, and 24-h urine specimens were taken at 24 weeks of age. Blood was collected via the medial canthus vein, and kidney tissue specimens were retained. Blood glucose, body weight, creatinine, urea, and 24-h urine protein levels were significantly higher in the BKS-DB group compared with those in the control group (*p* < 0.05) (Figure 8(a)). Compared with mice in the control group, HE staining results suggested that mice in the BKS-DB group showed more obvious detachment of tubular epithelial cells (TECs) or brush border and diffuse sclerosis of glomeruli. PAS staining results suggested that a plasma exudative fibrous cap was visible in mice in the BKS-DB group and dilatation of capillary shores. Masson staining results suggested that the renal tubules and mesangium of mice in the control group had no blue-stained tissue, and the collagen level of renal tubules and interstitium was significantly increased in mice in the BKS-DB group (Figure 8(b)).

### 3.8. Clinical Data Validation and Diagnostic Effects of Hub Genes in DKD

**Figure 9:**
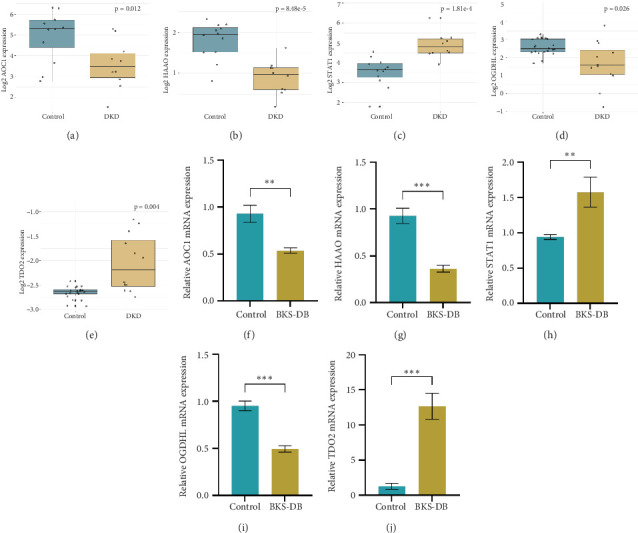
Validation and efficacy evaluation of key genes. Expression levels of (a) AOC1, (b) HAAO, (c) STAT1, (d) OGDHL, and (e) TDO2 mRNA in DKD patients compared with normal samples. The expression level of (f) AOC1, (g) HAAO, (h) STAT1, (i) OGDHL, and (j) TDO2 in DKD mice and normal mice was verified by qPCR. *n* = 6, ⁣^∗∗^*p* < 0.01, ⁣^∗∗∗^*p* < 0.001.

Nephrseq v5 showed that the mRNA expression levels of AOC1, HAAO, and OGDHL were downregulated in the kidneys of DKD patients compared with controls, while the mRNA levels of STAT1 and TDO2 were upregulated. The changes in the expression of all of these genes in DKD may be risk factors for the progression of DKD (Figures 9(a), 9(b), 9(c), 9(d), and 9(e)).

### 3.9. qPCR Validation of Hub Genes

RNA was extracted from 24-week-old BKS-DB mice and BKS/m mice. qPCR results showed that the mRNA expression levels of AOC1, HAAO, and OGDHL were downregulated in DKD mice, while the mRNA levels of STAT1 and TDO2 were upregulated in DKD mice when compared with the control group (*p* < 0.01) (Figures 9(f), 9(g), 9(h), 9(i), and 9(j)).

### 3.10. Immunohistochemistry and Western Blot Validation of Hub Genes

**Figure 10:**
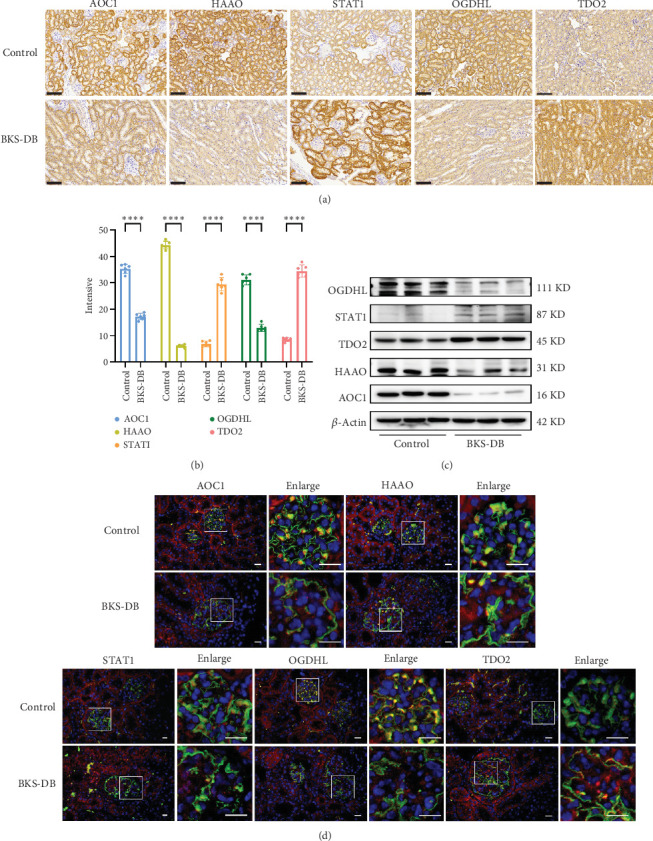
In vivo animal validation of hub genes. (a) Representative images of immunohistochemical staining of hub genes in kidney tissue sections of control and BKS-DB mice. Bar: 100 *μ*m. (b) Statistical analysis of immunohistochemical staining. *n* = 6. ⁣^∗∗∗∗^*p* < 0.0001. (c) Western blot verification of hub genes in kidney tissues of control and BKS-DB mice. *n* = 6. (d) Immunofluorescence colocalisation of hub genes in glomeruli verified by immunofluorescence in kidney tissue sections of control and BKS-DB mice. *n* = 6, bar: 20 *μ*m.

Immunohistochemistry results showed a significant decrease in the expression of AOC1, HAAO, and OGDHL (*p* < 0.0001) and a significant increase in the expression of STAT1 and TDO2 (*p* < 0.0001) in the BKS-DB group compared to the control group (Figure 10a,b). Western blot verified that the expression of the respective genes was consistent with the IHC (Figure 10c).

### 3.11. Immunofluorescence Validation of the Hub Gene

Immunofluorescence results showed that AOC1, HAAO, and STAT1 did not significantly colocalise with the podocyte-specific protein nephrin and were not significantly expressed in the glomerular region. OGDHL and TDO2 were seen to be expressed in the glomerular region. OGDHL expression was significantly downregulated in the glomerular region, and TDO2 expression significantly increased in the BKS-DB group compared to the control group (Figure 10d).

## 4. Discussion

DKD is a complex disease associated with numerous genetic loci identified in genome-wide association studies (GWASs) [16]. Although some progress has been made in the treatment of DKD, it remains a significant cause of end-stage renal disease (ESRD) [17]. Several studies have shown that TRP metabolism is closely related to the development of DKD. Zhu et al. [18] and others showed that TRP metabolism plays an important role in DKD mice, and it is a key factor leading to the disruption of microbial metabolic homeostasis in DKD mice [7].

In this study, for the first time, DKD and TRP metabolism data were combined to identify potential TRP-related biomarkers in DKD using bioinformatics methods. WGCNA was used to identify modular genes and take the intersection with differentially expressed genes and TRGs, and finally, five key genes, AOC1, HAAO, STAT1, OGDHL, and TDO2, were obtained. Their roles in the pathogenesis of DKD were confirmed by constructing a clinical prediction model. Moreover, their expression was verified by qPCR, Western blot, immunohistochemistry, and immunofluorescence analyses of the kidneys of 24-week-old BKS-DB mice.

AOC1 belongs to a protein-coding gene, a protein with specific physiological functions that oxidises putrescine, histamine, and related compounds [19]. Relevant studies have shown that polyamines are important in normal kidney development [20]. Changes in polyamine homeostasis affect branching morphogenesis in cultured mouse embryonic kidneys, and this effect is mediated by changes in AOC1 expression mediating polyamine catabolism, which plays a role in renal organogenesis through nephroblastoma protein (WT1)-dependent control [21]. The kidney maintains its homeostasis in the face of various forms of injury by downregulating polyamine synthesis and activating polyamine catabolic pathways [22]. However, relatively few studies have been conducted on AOC1 in CKD, and no relevant studies have been identified regarding AOC1 in the progression of DKD. In the present study, we observed that AOC1 was predominantly expressed in renal tubules. Notably, the expression of AOC1 showed a significant downregulation during the pathogenesis of DKD. However, further in-depth studies and explorations are needed on exactly how AOC1 downregulation mediates renal tubular injury and its role in DKD progression.

HAAO is an enzyme that catalyses the conversion of 3-hydroxy-octylaminobenzoic acid (3-OH-AA) to quinolinic acid (QA), which has been identified as a risk gene for hypospadias [23]. Wang et al. [24] found that the expression of HAAO was significantly reduced in diabetic kidneys, which may be responsible for the reduced urinary QA/3-OH-AA levels. In addition, a recent study has shown that 3-OH-AA is a major immunomodulatory metabolite that regulates vascular inflammation and lipid levels [25]. In recent years, the role of immunomodulatory effects in the development and progression of DKD has attracted much attention, and the study found that HAAO was significantly downregulated in DKD using bioinformatics screening and validated by DKD animal tissue samples. Based on this, we hypothesised that HAAO may be an important immunomodulatory metabolite in the progression of DKD and is expected to be an important target for DKD immunotherapy.

STAT1, as a member of the STAT family, can mediate various cellular functions in response to stimulation by cytokines, growth factors, and hormones such as IFN and IL-6 [26]. Relevant studies have shown that the JAK-STAT pathway plays an important role in the progression of DKD, specifically by promoting the expression of inflammatory factors and inducing the activation of inflammatory cells [27]. Not only that, the phosphorylation of STAT1 also has an inhibitory effect, which can inhibit the M1 phenotypic transformation of macrophages and thus has an inhibitory effect on the progression of DKD [28]. Recently, a study by Li et al. [29] found that high glucose-induced STAT activation increases the expression of proinflammatory and profibrotic factors in glomerular and tubular cells while leading to the infiltration of circulating inflammatory cells, which amplifies and perpetuates the inflammatory process in the kidneys and promotes the development of DKD. In line with previous findings, the present study, using bioinformatics methods, found that STAT1 expression was significantly upregulated in DKD patients and was validated using tissues from 24-week-old DKD mice. Based on this, STAT1 may be an important target in regulating the progression of DKD and play a key role in regulating inflammation and fibrosis.

Oxyglutarate dehydrogenase (OGDHL), which can encode a rate-limiting Krebs cycle enzyme, is currently the subject of research focusing on neurodevelopmental disorders, tumours, and other related diseases and is thought to be closely associated with highly variable manifestations of neurological and neurodevelopmental disorders [30]. Downregulation of OGDHL expression alters mitochondrial function, contributing to increased cellular proliferation, which can lead to the development and progression of several cancers [31]. Specifically, in a diabetic nephropathy model, glomerular podocyte injury involves mitochondrial dysfunction, including irregular dynamics [32], and it can be inferred that OGDHL plays a potential role in developing DKD. In addition, OGDHL, as an isoform of 2-OGDHL, regulates glucose and glutamate degradation [33]. From this, we hypothesise that OGDHL plays an important role in mitochondrial oxidative phosphorylation and energy metabolism. There are relatively few studies on OGDHL in DKD. In this study, we found that the expression of OGDHL was significantly downregulated in the DKD group compared with the control group by comparison. Meanwhile, immunofluorescence colocalisation studies using OGDHL and nephrin revealed that the difference in OGDHL expression between the two groups was more pronounced on glomerular podocytes. The downregulation of OGDHL may be associated with the progression of DKD, and this effect may be related to podocyte injury.

TRP 2,3-dioxygenase (TDO2) is one of the enzymes involved in the metabolism of TRP through the Kyn pathway, which accounts for approximately 95% of the metabolic process of TRP [34]. TDO2 can inhibit inflammation and maintain immune homeostasis through TRP starvation [35]. This group of enzymes is directly or indirectly involved in developing various diseases, including inflammatory diseases, cancer, diabetes, and psychiatric disorders [36]. Zhong et al. [37] found a compensatory increase in TDO due to a deficiency of the initiating and rate-limiting enzyme of the TRP-degrading Kyn pathway, indoleamine-2,3-dioxygenase 1 (IDO1), and alleviated CCl4-induced hepatic fibrosis. They hypothesised that increased TDO would consume more TRP, thereby reducing the number of CD8+ T cells that might attack damaged hepatocytes. Recently, Qin and Zhou [38] found that knockdown of TDO2 in NAFLD mice inhibited hepatic lipid deposition and hepatic fibrosis and reduced the expression of markers related to hepatic lipid deposition and fibrosis in PA-treated primary hepatocytes by inactivating the NF-*κ*B pathway. Given the important role of TDO2 in the progression of hepatic fibrosis, we speculated that it may also be an important gene involved in renal fibrosis in DKD. Immunohistochemical analysis of DKD mice revealed that the expression of TDO2 was significantly elevated on renal tubules and glomeruli compared with the healthy group. Moreover, by fluorescence colocalisation of TDO2 with nephrin, it was also suggested that the expression of TDO2 was significantly elevated in DKD renal podocytes. However, further studies need to confirm whether inhibiting the expression of TDO2 can improve DKD renal fibrosis and the related mechanisms.

NK cells can also damage renal TECs and trigger renal ischaemia–reperfusion injury (IRI) in the absence of T- and B-cell involvement [39–41]. Lan et al. [42] found, through the analysis of differences in immune cell infiltration by pivotal DE-CRGs, which are obtained by hybridising DEGs with copper death–related genes (CRGs), that regular and DKD rats' resting NK cells were significantly different, with upregulation of resting NK cells in DKD rats. In the present study, we found that AOC1, HAAO, and STAT1 were significantly associated with activated NK cell infiltration, and all three genes were predominantly expressed in the renal tubules. Specifically, AOC1 and HAAO expression was significantly downregulated in DKD, whereas STAT1 expression was significantly upregulated in patients with DKD. Notably, the involvement of AOC1, HAAO, and STAT1 in regulating NK cells has not been previously reported, which requires further in-depth studies.

DKD is considered a comprehensive inflammatory disorder regulated primarily by T cells [43]. *γ δ* T cells act as a distinct subpopulation of T cells [44], where specific subpopulations of *γ δ* TCR+cells, such as CD27-CD44hi and CD27+CD44lo, are increased in number in diabetic nonobese diabetic (NOD) mice [45]. Our understanding of the specific mechanism of action of *γ δ* T cells in DKD is limited. Studies have reported that in diabetic patients, the number of CD4+T cells is positively correlated with proteinuria [46]; in crescentic glomerulonephritis (cGN), glomerular injury depends on CD4+T cells [47], and in agreement with the related studies, the present study found that there was a significant difference between the resting CD4+ T cells on the glomeruli in the healthy group and the DKD group (*p* < 0.05). Therefore, we hypothesised that CD4+ T cells are involved in developing DKD. Monocytes, as a major cellular component of the innate immune system, are associated with complications and progression of CKD [48]. In this study, we found that the expression of TDO2 was significantly elevated on both renal tubules and glomeruli, but its relationship with immune cell infiltration and whether inhibiting the expression of TDO2 can improve renal fibrosis and related mechanisms in DKD need to be confirmed by further studies.

This study has several limitations. The study results are based on public databases and lack detailed clinical information, such as the specific severity of the kidneys and survival data. Therefore, further investigation of prospective clinical data is essential to validate the results of this study. In addition, the clinical data provided by Nephrseq v5 has a limited sample size for inclusion, and the differential expression of key genes in DKD patients needs to be validated by a larger independent cohort. The present study, which only used bioinformatics analysis to explore the relationship between the pivotal genes and immune cell infiltration, is insufficient for further validation and in-depth exploration in vivo and in vitro. Finally, the present study only launched the expression difference analysis for five key genes related to TRP metabolism in the healthy and DKD groups, and more studies are needed to confirm the targets and specific mechanisms of the role of these genes in the progression of DKD.

## 5. Conclusion

We identified five key genes (AOC1, HAAO, STAT1, OGDHL, and TDO2) closely associated with TRP metabolism in DKD patients and may be critical for developing DKD. This study provides insights into the pathogenesis of DKD.

## Figures and Tables

**Table 1:** Information on datasets included in the study.

**Dataset ID**	**Platform**	**Tissue**	**Samples (number)**			**Attribute**
			**Total**	**DKD**	**Control**	
GSE30528	GPL571	Glomeruli	22	9	13	Training
GSE30529	GPL571	Tubuli	22	10	12	Training
GSE104954	GPL22945	Tubuli	25	7	18	Validation
GSE142025	GPL20301	Whole kidney	36	27	9	Validation

## Data Availability

The data that support the findings of this study are available from the corresponding author upon reasonable request.

## Supporting Information

Supporting Information Additional supporting information can be found online in the Supporting Information section. Table S1: Information on genes related to tryptophan metabolism. Table S2: qPCR primer sequence.
