# Supplementary material for: Identification and Validation of Tryptophan Metabolism–Related Genes in Diabetic Kidney Disease and Construction of a Clinical Prediction Model
Source: J Diabetes Res. 2025 May 8;2025:2736801. doi: 10.1155/jdr/2736801 (PMC12081153; doi:10.1155/jdr/2736801)
Supplement: Supporting Information — Additional supporting information can be found online in the Supporting Information section. Table S1: Information on genes related to tryptophan metabolism. Table S2: qPCR primer sequence. [file 2736801.f1.docx]

**Suppplement table 1 Information on genes related to tryptophan metabolism**

| **GeneCards Symbol** | **Gene Name** |
| --- | --- |
| **AADAT** | **Aminoadipate Aminotransferase** |
| **AANAT** | **Aralkylamine N-Acetyltransferase** |
| **ACAT1** | **Acetyl-CoA Acetyltransferase 1** |
| **ACAT2** | **Acetyl-CoA Acetyltransferase 2** |
| **ACMSD** | **Aminocarboxymuconate Semialdehyde Decarboxylase** |
| **AFMID** | **Arylformamidase** |
| **AHR** | **Aryl Hydrocarbon Receptor** |
| **ALDH1B1** | **Aldehyde Dehydrogenase 1 Family Member B1** |
| **ALDH2** | **Aldehyde Dehydrogenase 2 Family Member** |
| **ALDH3A2** | **Aldehyde Dehydrogenase 3 Family Member A2** |
| **ALDH7A1** | **Aldehyde Dehydrogenase 7 Family Member A1** |
| **ALDH8A1** | **Aldehyde Dehydrogenase 8 Family Member A1** |
| **ALDH9A1** | **Aldehyde Dehydrogenase 9 Family Member A1** |
| **AOC1** | **Amine Oxidase Copper Containing 1** |
| **AOX1** | **Aldehyde Oxidase 1** |
| **ASMT** | **Acetylserotonin O-Methyltransferase** |
| **CAT** | **Catalase** |
| **CYP1A1** | **Cytochrome P450 Family 1 Subfamily A Member 1** |
| **CYP1A2** | **Cytochrome P450 Family 1 Subfamily A Member 2** |
| **CYP1B1** | **Cytochrome P450 Family 1 Subfamily B Member 1** |
| **DDC** | **Dopa Decarboxylase** |
| **DLD** | **Dihydrolipoamide Dehydrogenase** |
| **ECHS1** | **Enoyl-CoA Hydratase, Short Chain 1** |
| **EHHADH** | **Enoyl-CoA Hydratase And 3-Hydroxyacyl CoA Dehydrogenase** |
| **GCDH** | **Glutaryl-CoA Dehydrogenase** |
| **GOT2** | **Glutamic-Oxaloacetic Transaminase 2** |
| **HAAO** | **3-Hydroxyanthranilate 3,4-Dioxygenase** |
| **HADH** | **Hydroxyacyl-CoA Dehydrogenase** |
| **HADHA** | **Hydroxyacyl-CoA Dehydrogenase Trifunctional Multienzyme Complex Subunit Alpha** |
| **IDO1** | **Indoleamine 2,3-Dioxygenase 1** |
| **IDO2** | **Indoleamine 2,3-Dioxygenase 2** |
| **IL4I1** | **Interleukin 4 Induced 1** |
| **INMT** | **Indolethylamine N-Methyltransferase** |
| **KMO** | **Kynurenine 3-Monooxygenase** |
| **KYAT1** | **Kynurenine Aminotransferase 1** |
| **KYAT3** | **Kynurenine Aminotransferase 3** |
| **KYNU** | **Kynureninase** |
| **MAOA** | **Monoamine Oxidase A** |
| **MAOB** | **Monoamine Oxidase B** |
| **OGDH** | **Oxoglutarate Dehydrogenase** |
| **OGDHL** | **Oxoglutarate Dehydrogenase L** |
| **SLC36A4** | **Solute Carrier Family 36 Member 4** |
| **SLC3A2** | **Solute Carrier Family 3 Member 2** |
| **SLC7A5** | **Solute Carrier Family 7 Member 5** |
| **STAT1** | **Signal Transducer And Activator Of Transcription 1** |
| **TDO2** | **Tryptophan 2,3-Dioxygenase** |
| **TPH** | **Tryptophan Hydroxylase** |
| **TPH1** | **Tryptophan Hydroxylase 1** |
| **TPH2** | **Tryptophan Hydroxylase 2** |
| **WARS1** | **Tryptophanyl-TRNA Synthetase 1** |
| **WARS2** | **Tryptophanyl TRNA Synthetase 2, Mitochondrial** |

**Suppplement table 2 Primer sequence**

| **Primer** | **primer sequence（5'to3'）** |
| --- | --- |
| AOC1-F(mus） | GACACTACTGGCTTCTCATTCCTA |
| AOC1-R(mus） | CCTGGACATCTGTGCTGCTA |
| HAAO-F(mus） | GCAACAAGCTTATGCACCAGG |
| HAAO-R(mus） | GCTGGTAAAACACCTCCTCAC |
| STAT1-F(mus） | TGACGACCCTAAGCGAACTG |
| STAT1-R(mus） | AGACATGGGAAGCAGGTTGT |
| OGDHL-F(mus） | GGTGTCAGGAGGAACATAAGAAC |
| OGDHL-R(mus） | CAGATTGAAGGCAGTATCCAGAA |
| TDO2-F(mus） | GGCTATCATTACCTGCGTTCAA |
| TDO2-R(mus） | TCGGCTGTGTAAAGGAATTTGT |
